# Burrowing Criteria and Burrowing Mode Adjustment in Bivalves to Varying Geoenvironmental Conditions in Intertidal Flats and Beaches

**DOI:** 10.1371/journal.pone.0025041

**Published:** 2011-09-21

**Authors:** Shinji Sassa, Yoichi Watabe, Soonbo Yang, Tomohiro Kuwae

**Affiliations:** 1 Soil Mechanics and Geo-Environment Research Group, Port and Airport Research Institute, Yokosuka, Japan; 2 Coastal and Estuarine Environment Research Group, Port and Airport Research Institute, Yokosuka, Japan; National Institute of Water & Atmospheric Research, New Zealand

## Abstract

The response of bivalves to their abiotic environment has been widely studied in relation to hydroenvironmental conditions, sediment types and sediment grain sizes. However, the possible role of varying geoenvironmental conditions in their habitats remains poorly understood. Here, we show that the hardness of the surficial intertidal sediments varies by a factor of 20–50 due to suction development and suction-induced void state changes in the essentially saturated states of intertidal flats and beaches. We investigated the response of two species of bivalves, *Ruditapes philippinarum* and *Donax semigranosus*, in the laboratory by simulating such prevailing geoenvironmental conditions in the field. The experimental results demonstrate that the bivalve responses depended strongly on the varying geoenvironmental conditions. Notably, both bivalves consistently shifted their burrowing modes, reducing the burrowing angle and burial depth, in response to increasing hardness, to compensate for the excessive energy required for burrowing, as explained by a proposed conceptual model. This burrowing mode adjustment was accompanied by two burrowing criteria below or above which the bivalves accomplished vertical burrowing or failed to burrow, respectively. The suitable and fatal conditions differed markedly with species and shell lengths. The acute sensitivities of the observed bivalve responses to geoenvironmental changes revealed two distinctive mechanisms accounting for the adult–juvenile spatial distributions of *Ruditapes philippinarum* and the behavioral adaptation to a rapidly changing geoenvironment of *Donax semigranosus*. The present results may provide a rational basis by which to understand the ensuing, and to predict future, bivalve responses to geoenvironmental changes in intertidal zones.

## Introduction

Burrowing of macroinfauna in intertidal sediments constitutes an intrinsic part of their basic living activities and is as fundamental as foraging and breeding. Burrowing performance may thus be an important factor in determining the distribution of species [1]–[6] and in forming the macroinfaunal community of intertidal zones [7]. The burrowing performance of individual species may be affected by their traits (morphology, body size, etc.) [8],[9], sediment grain size [10]–[12], temperature [13]–[15], salinity [16], geochemistry [17], and intertidal geomorphodynamics in relation to swash climate [18]. Small species with streamlined shapes may be best adapted to the dynamic swash conditions [8].

For the macroinfauna living in intertidal sediments, physical processes in the sediments that vary markedly in space and time have a critical influence on their activities, particularly considering that burrowing represents physical action in sediments [19],[20]. However, to date, the physical environments affecting the infaunal activities and distributions have been mainly associated with the physical processes of fluids above the sediments, such as tides, waves, and currents [2],[3],[8],[9],[21]–[24]. Swash-induced hydrodynamics has also been considered to govern the adaptation of macroinfauna at exposed sandy beaches [25]. The sediment types, such as sand and mud, have been related to macroinfaunal distributions [4],[6]. However, because of the lack of general understanding of the physical processes involved in intertidal sediments [20], the response of macroinfauna to variations in their geophysical environment remains much less known, in contrast to their response to variations in their hydroenvironment with given sediments.

Recently, Sassa & Watabe [19] demonstrated that the dynamics of suction, that is, negative pore water pressure relative to atmospheric air pressure, in association with tide-induced groundwater level fluctuations, plays a substantial role in controlling the geophysical environment of habitats. Namely, the suction dynamics bring about the temporal and spatial evolutions of voids, stiffness, and hardness of intertidal flat sediments. The effects of the suction dynamics have also been shown to play a crucial role in intertidal flat geomorphodynamics [26], and in forming the intertidal flat stratigraphy of sandy, muddy, and sand–mud layered sediments [27]. Understanding such salient geophysics involved in intertidal sediments has facilitated close investigation of the linkage between the geophysical environment and the ecology of intertidal flats. This has revealed the threshold, optimum, and critical geoenvironmental conditions for the burrowing activity of sand bubbler crab, *Scopimera globosa* (Crustacea: Ocypodidae) [20], and the close link between the suction-induced temporal variation in sediment hardness and the foraging behavior of shorebird, *Calidris alpina* [28].

In the present study, we demonstrate a crucial role of varying geoenvironmental conditions in the response of bivalves with different shapes and sizes. Although the response of bivalves to their abiotic environment has been widely studied in relation to the hydroenvironmental conditions and sediment types and sediment grain sizes [2]–[4],[6],[8],[10]–[12],[21]–[25], the possible role of the varying geoenvironmental conditions in their habitats remains poorly understood. Here, we studied the linkage between the response of two common species of bivalves, *Ruditapes philippinarum* (Mollusca: Veneridae) and *Donax semigranosus* (Mollusca: Donacidae), and the relevant geophysical environmental conditions. For this purpose, we utilized a new high-resolution laboratory measurement system, in conjunction with the field testing apparatus. Burrowing of bivalves is essentially different in form from the burrowing of crabs and worms, since bivalves do not typically create burrows (cavities). For bivalves that have specific siphon lengths [29], vertical burrowing [30] or self-burial, which has a deeper center of gravity than any inclined burrowing, can maximize the stability of bivalves in sediments, thus minimizing the risk of surface transport, i.e., sweeping away [31], and exposure to predators [32] and direct sunlight [33]. Our controlled laboratory experiments, together with our field surveys and proposed conceptual model, revealed the existence and mechanics of novel burrowing criteria and burrowing mode adjustment to varying geoenvironmental conditions in juvenile to adult *R. philippinarum* and *D. semigranosus*. These have led to substantial new insights into the distributions and adaptations of the bivalves in intertidal flats and beaches.

## Materials and Methods

### Materials

The bivalve *Ruditapes philippinarum*, the Manila clam, inhabits intertidal sandy flats and is commercially important to the fishery industry not only in Japan [34],[35] but also in other countries [36],[37]. The bivalve *Donax semigranosus* inhabits the intertidal zones of exposed sandy beaches in Japan. *Donax* species that belong to Donacidae have been commonly used for burrowing studies, focusing on the influences of swash climates and sediment grain sizes [8],[11],[12].

Our study sites in Japan involved two intertidal sandy flats as the habitats of *R. philippinarum* and two sandy beaches as the habitats of *D. semigranosus*. We performed field surveys from 2009 to 2010 during the spring low tides at the Nojima tidal flat (N35°19′, E139°38′; Mar. 2009, Sep. 2010), the Shirakawa tidal flat (N32°47′, E130°35′; Sep. 2009), Yuigahama beach (N35°18′, E139°32′; Sep. 2010), and Kujyukuri beach (N35°31′, E140°27′; May 2009). The intertidal sediments at the four study sites were essentially composed of fine-grained sands with median grain diameters in the range D_50_ = 0.14–0.27 mm. The silt and clay contents were less than 25% at the intertidal flats and below 1% at the sandy beaches.

For the burrowing experiments in the laboratory, we collected adult to juvenile bivalves *R. philippinarum* at three intertidal flats: Nojima, Banzu (N35°24′, E139°54′) and Furenko (N43°17′, E145°22′) tidal flats. The shell lengths L ranged from 2.5 mm to 52 mm, and the individual wet weights *w* ranged from 0.004 g to 31.9 g. We confirmed that *w* was a single function of L such that *w*(L) = 1.860×10^−4^·L^3.043^, r^2^ = 0.99, p<0.0001, n = 1083. We also collected *D. semigranosus* at Yuigahama beach with L = 3.7 mm to 15 mm and *w* = 0.01 g to 0.8 g, yielding the relationship: *w*(L) = 1.853×10^−4^·L^3.026^, r^2^ = 0.963, p<0.0001, n = 604.

### Methods

Let us first list some relevant physical quantities that represent the geophysical states of intertidal sediments.

Suction, 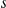, means the tension of moisture in the sediment [38] and is defined by
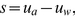
(1)where 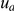 is the atmospheric air pressure, and 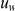 is the pore water pressure in the sediment. By definition, suction is equal to zero at the groundwater level.

The void state of the sediment is represented by void ratio *e*, which is related to the sediment porosity *n*:
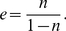
(2)The state of sediment packing, such as dense or loose, can be denoted by the sediment relative density *D_r_*:
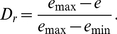
(3)


For a given sediment, the maximum void ratio 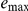 represents the loosest possible packing, and the minimum void ratio 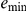 represents the densest possible packing [39]. Thus, the 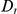 value is a normalized index by which to assess the packing states of sandy sediments.

**Figure 1:**
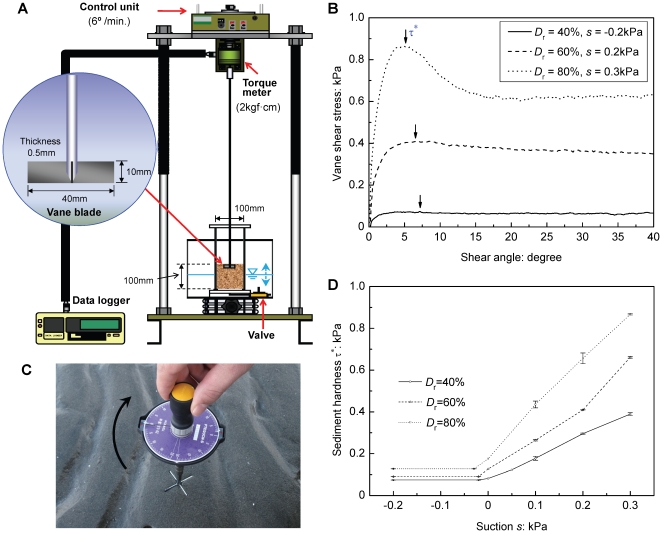
Sediment hardness as assessed by the vane shear strength. (A) High-resolution vane shear testing system in the laboratory. Sediments with prescribed sediment relative densities *D*_r_ were formed in an acrylic cylindrical chamber set in a larger water tank. Suctions *s* at the level of the sediment surface were varied by changing the water level in the tank. The vane shear testing was performed by inserting and rotating the vane blade in the uppermost layer of the given sediment. (B) Results of the vane shear testing. The peak value of the measured vane shear stresses represents the vane shear strength τ*, namely the sediment hardness. (C) Field vane shear testing apparatus for surficial sediments. The apparatus directly measures τ*. (D) Sediment hardnesses simulated in the laboratory as functions of suctions and sediment relative densities of the intertidal sediments taken at the Nojima tidal flat. Data in (D) were obtained using both apparatuses shown in (A) and (C) and represent mean values ± SE.

The hardness of surficial intertidal sediments can be assessed by the vane shear strength [19],[20],[28],[40]. An important feature of the vane shear testing is that it can evaluate an in-situ undisturbed state of the sediment hardness by inserting a very thin vane blade into the surficial sediment and measuring the maximum resistance τ* of the sediment to horizontal shearing due to rotating the vane blade (Figure 1A,B,C). Sediment hardness as assessed by the vane shear strength τ* has been shown to govern the development of burrows of sand bubbler crabs [20] and to be closely linked with the foraging mode shift by shorebirds [28]. A difference with these previous studies was that here we measured the hardness of the uppermost sediment by adopting a vane blade of 10 mm depth rather than 40 mm depth, in order to cope with the observed higher sensitivity of the bivalve responses to the varying geoenvironmental conditions, as described later in this paper.

We measured the distributions of suction and hardness of the surficial sediments in the four intertidal flats and intertidal zones of exposed sandy beaches: Nojima and Shirakawa tidal flats and Yuigahama and Kujyukuri beaches. Here, suctions were measured using tensiometers [19]. Additionally, at each site of the Yuigahama and Kujyukuri beaches, continuous measurements of suctions were performed by installing multiple tensiometers along the cross-shore survey transect in order to investigate the swash-induced suction dynamics in the intertidal zones of the exposed sandy beaches.

We compared the above field results with the laboratory measurements. In the laboratory, sediment deposits with three different states of packing at *D*_r_ = 40%, 60%, and 80% were formed in a transparent cylindrical chamber, shown in Figure 1A, by using the intertidal sediments taken from the Nojima tidal flat. Suctions *s* at the level of the sediment surface were varied by changing the water level above and the groundwater level in the sediment by using the system in Figure 1A. Both the laboratory and field apparatuses (Figure 1A,C) were used to measure the hardnesses of the sediments formed. Figure 1D shows that both measurements were precisely performed, as indicated by the small error bars. The sediment hardness τ* increased with increasing suction *s* and sediment relative density *D*_r_, and depended only on sediment relative density *D*_r_ under negative suctions (submerged condition).

The protocols of the burrowing experiments for *R. philippinarum* and *D. semigranosus* are shown in Tables 1 and 2. Here, the prescribed different suctions *s* and relative densities *D*_r_ gave rise to different states of the sediment hardness τ*, as described above.

**Table 1:** Protocol for burrowing experiments on *Ruditapes philippinarum*.

W.L./G.W.L.	*s*	*D*_r_	τ^*^	L				
mm	kPa	%	kPa	4∼6 mm	10∼12 mm	19∼21 mm	29∼31 mm	49∼51 mm
20 (40)^a^	−0.2 (−0.4)	40	0.07	n = 8, *a* = 8	n = 4, *a* = 4	n = 4, *a* = 4	n = 3, *a* = 2, *b* = 1	n = 4, *a* = 1, *b* = 3
0	0	40	0.08			n = 4, *a* = 4	n = 3, *a* = 2, *b* = 1	
20 (40)^a^	−0.2 (−0.4)	60	0.09	n = 8, *a* = 7, *b* = 1	n = 4, *a* = 4	n = 4, *a* = 4	n = 4, *a* = 2, *b* = 2	n = 4, *b* = 4
−5	0.05	40	0.12		n = 4, *a* = 2, *b* = 2	n = 4, *a* = 1, *b* = 3		
20 (40)^a^	−0.2 (−0.4)	80	0.13	n = 8, *a* = 1, *b* = 7	n = 4, *a* = 4	n = 4, *a* = 2, *b* = 2	n = 4, *a* = 2, *b* = 2	n = 4, *b* = 2, *c* = 2
0	0	60	0.13	n = 8, *a* = 1, *b* = 7	n = 4, *a* = 2, *b* = 2	n = 4, *b* = 4	n = 4, *b* = 3, *c* = 1	
0	0	80	0.18	n = 8, *b* = 5, *c* = 3	n = 4, *a* = 1, *b* = 3	n = 4, *b* = 2, *c* = 2	n = 4, *a* = 1, *b* = 1, *c* = 2	n = 4, *c* = 4
−10	0.1	40	0.18	n = 8, *c* = 8	n = 8, *b* = 7, *c* = 1	n = 4, *b* = 2, *c* = 2	n = 4, *c* = 4	
−5	0.05	60	0.20		n = 8, *b* = 7, *c* = 1	n = 4, *b* = 3, *c* = 1		
−8	0.08	60	0.24		n = 8, *b* = 5, *c* = 3			
−15	0.15	40	0.24		n = 8, *c* = 8	n = 4, *b* = 1, *c* = 3		
−3	0.03	80	0.25		n = 8, *b* = 1, *c* = 7			
−10	0.1	60	0.26		n = 8, *c* = 8	n = 4, *c* = 4		
−20	0.2	40	0.30			n = 4, *c* = 4	n = 4, *c* = 4	
−5	0.05	80	0.31		n = 8, *c* = 8	n = 4, *c* = 4		
−30	0.3	40	0.39			n = 4, *c* = 4		
−20	0.2	60	0.41			n = 4, *c* = 4		
−10	0.1	80	0.44			n = 4, *c* = 4		
−30	0.3	60	0.66			n = 4, *c* = 4		

W.L. : Water level, G.W.L. : Groundwater level, *s* : Suction, *D*_r_ : Sediment relative density, τ* : Sediment hardness, L : Shell length.

Air temp. : 20.2±0.2°C, Water temp. : 19.1±0.4°C, Salinity : 27 psu.

a( ): Case of L = 30, 50 mm.

Symbols *a*, *b*, and *c* denote the observed results. The symbol *a* means that the individual completed vertical burrowing (z* = −1, *θ* = 90±10°). The symbol *b* means that the individual exhibited inclined burrowing (0<*θ*<80°) and/or partial burrowing (−1<z*<0). The symbol *c* means that the burrowing was impossible (z* = 0 and *θ* = 0). Air and water temperatures are mean values ± SE.

**Table 2 pone-0025041-t002:** Protocol for burrowing experiments on *Donax semigranosus*.

W.L./G.W.L.	*s*	*D*_r_	τ^*^	L
mm	kPa	%	kPa	10∼11 mm
20	−0.2	40	0.07	n = 4, *a* = 4
20	−0.2	60	0.09	n = 4, *a* = 4
−5	0.05	40	0.12	n = 4, *a* = 4
20	−0.2	80	0.13	n = 4, *a* = 4
0	0	60	0.13	n = 4, *a* = 4
0	0	80	0.18	n = 4, *a* = 1, *b* = 3
−10	0.1	40	0.18	n = 4, *a* = 2, *b* = 2
−5	0.05	60	0.20	n = 4, *a* = 1, *b* = 3
−15	0.15	40	0.24	n = 4, *b* = 4
−10	0.1	60	0.26	n = 4, *b* = 3, *c* = 1
−20	0.2	40	0.30	n = 4, *b* = 2, *c* = 2
−5	0.05	80	0.31	n = 4, *b* = 3, *c* = 1
−15	0.15	60	0.34	n = 4, *b* = 2, *c* = 2
−25	0.25	40	0.34	n = 4, *b* = 1, *c* = 3
−7	0.07	80	0.36	n = 4, *c* = 4
−17	0.17	60	0.37	n = 4, *c* = 4
−30	0.3	40	0.39	n = 4, *c* = 4

W.L. : Water level, G.W.L. : Groundwater level, *s* : Suction, *D*_r_ : Sediment relative density, τ* : Sediment hardness, L : Shell length.

Air temp. : 20.4±0.2°C, Water temp. : 18.6±0.2°C, Salinity : 27 psu.

Symbols *a*, *b*, and *c* denote the observed results. The symbol *a* means that the individual completed vertical burrowing (z* = −1, *θ* = 90±10°). The symbol *b* means that the individual exhibited inclined burrowing (0<*θ*<80°) and/or partial burrowing (−1<z*<0). The symbol *c* means that the burrowing was impossible (z* = 0 and *θ* = 0). Air and water temperatures are mean values ± SE.

**Figure 2:**
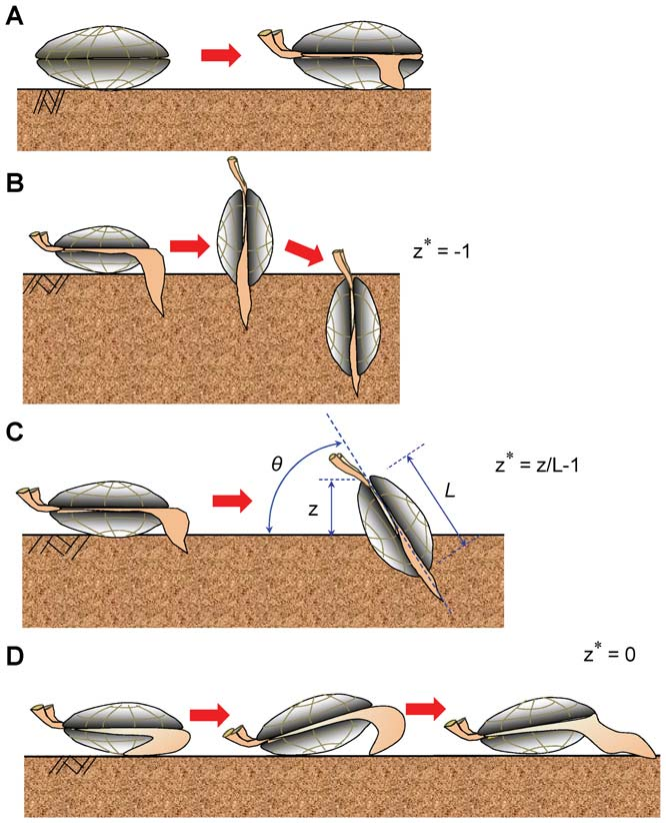
Definition of burrowing behavior for *Ruditapes philippinarum* and *Donax semigranosus*. (A) Starting condition for each individual in the burrowing experiments. (B) Observed typical processes of complete vertical burrowing. (C) Definition of normalized burrowing depth z^*^ and burrowing angle *θ*, showing the state of inclined and partial burrowing. (D) Observed typical processes where burrowing was impossible, showing bending of foot and rebounding from sediment surface. The bivalve shape represents that of *Ruditapes philippinarum*.

For the burrowing experiments, we used the juvenile to adult bivalves *R. philippinarum* with L = 5 mm, 10 mm, 20 mm, 30 mm, and 50 mm, and *D. semigranosus* with L = 10 mm. We observed the burrowing responses of these bivalves to the prescribed different states of the geophysical environmental conditions shown in Tables 1 and 2. In each experiment, we measured the burrowing depth, burrowing angle, and burrowing time for a period of six hours after the bivalve touched the sediment surface with its foot (Figure 2A). This starting condition was essential in obtaining consistent results, after the bivalves showed their “intention” to burrow in the sediments.

In all experiments, the air temperature, the water temperature, and the salinity of the water and pore water were kept essentially constant at 20 to 21°C, 19 to 20°C, and 27 psu, respectively (Tables 1 and 2). Prior to the experiments, the bivalves were maintained in the laboratory under aerated fresh seawater in the intertidal sediments for over one month to ensure that any endogenous physiological rhythms were abolished [41].

The burrowing characteristics observed in each case are summarized in Tables 1 and 2 by using three symbols, *a*, *b* and *c*. With reference to Figure 2B, C, and D, the symbol *a* represents the situation where an individual bivalve burrowed essentially vertically (*θ* = 90±10°) and buried itself underneath the sediment surface (z^*^ = −1). Here, the burrowing angle *θ* denotes the angle at the final stage of burrowing. The symbol *b* refers to the situation where an individual bivalve exhibited inclined burrowing (0<*θ*<80°) and/or resulted in partial burrowing (−1<z^*^<0). The symbol *c* indicates the situation where burrowing was physically impossible (z^*^ = 0 and *θ* = 0). Under such a situation, the bivalves often bent their feet and rebounded from the sediment surface, as shown in Figure 2D.

In cases where all the bivalves completed the burrowing, either vertical or inclined, the average burrowing times ranged widely from 1.5 min to 105 min for *R. philippinarum* and from 18 s to 51 s for *D. semigranosus*.

### Statistical analyses

We used a generalized linear model (GLM) with a binomial error distribution to examine the effect of species, shell length, and sediment hardness on burrowing depth z^*^ and burrowing angle *θ*. *A priori* selection of candidate models was based on the principle of parsimony and scientific plausibility [42]. We fitted the global model with species, z^*^ and *θ*, and the second order interactions. We used Akaike's Information Criterion (AIC) to compare the fits of candidate models. The best fitting model has the smallest AIC.

We also used a generalized additive model (GAM) with a binomial error distribution to examine how burrowing response co-varied with shell length and sediment hardness in *R. philippinarum*. We selected shell length and sediment hardness as the explanatory variables and binary data for vertical burrowing and burrowing mode shift (*a* and *b*) and failure (*c*) in Table 1 as the response variable for the model. We performed all statistical analyses using R 2.1.1.

## Results

### Variation in geophysical environment of habitats in intertidal flats and beaches

**Figure 3:**
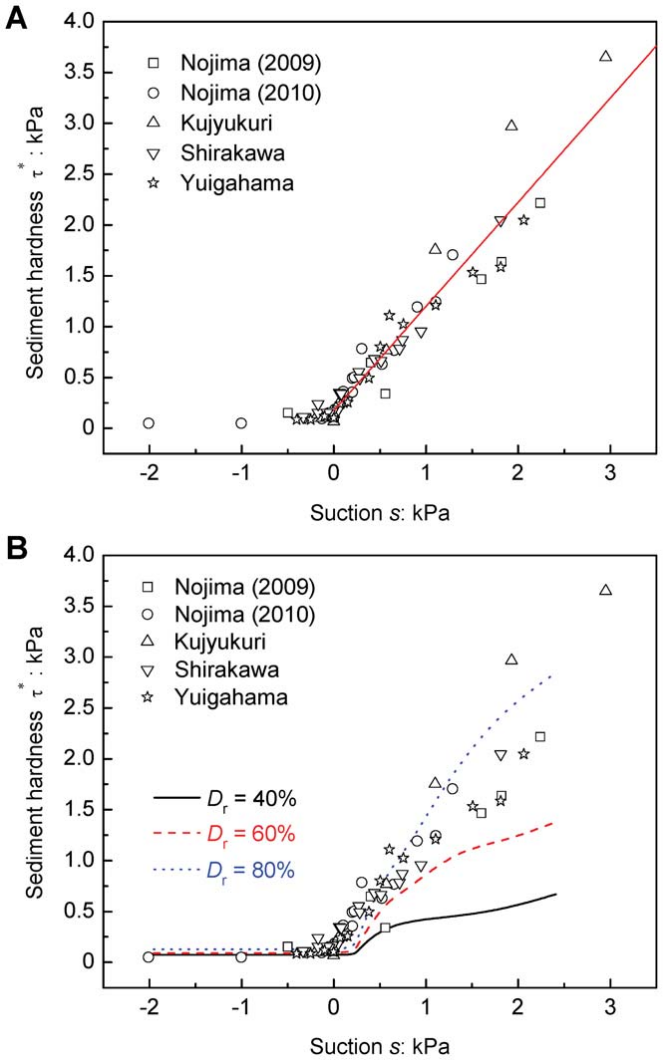
Interrelationships between suction, sediment relative density, and sediment hardness in the laboratory and the field. (A) Measured relationships between sediment hardness τ* and suction *s* at four intertidal flats and beaches. Both of τ* and *s* were measured during spring low tides at the Nojima and Shirakawa tidal flats and during spring low tides when the swash retreated in the intertidal zones of Kujyukuri and Yuigahama beaches. All the measured data at the Nojima tidal flat (n = 23, r^2^ = 0.912, p<0.0001), the Shirakawa tidal flat (n = 20, r^2^ = 0.967, p<0.0001), Kujyukuri beach (n = 12, r^2^ = 0.976, p<0.0001), and Yuigahama beach (n = 14, r^2^ = 0.932, p<0.0001), fell on a unique relationship τ* = 1.02462*s*+0.17557 (n = 69, r^2^ = 0.914, p<0.0001). (B) Comparison between the field and laboratory data. The symbols represent the field data shown in (A). The three different lines represent the laboratory data for the three different sediment relative densities *D*_r_ = 40%, 60%, 80%, and cover higher suction ranges than those shown in Figure 1D.

The hardness of the surficial intertidal sediments varied markedly at the Nojima tidal flat, the Shirakawa tidal flat, Kujyukuri beach, and Yuigahama beach (Figure 3A). Indeed, the sediment hardness had strong correlations with suctions at all four intertidal flats and beaches (r^2^>0.9, p<0.0001). At the Nojima flat, the sediment hardness τ* was only 0.05 kPa at negative suctions *s*<−1 kPa; however, it reached as high as 2.2 kPa at a suction of 2.2 kPa. This corresponds to a 44-fold increase in hardness of the surficial sediments. The other three sites exhibited similar variations, showing 20- to 50-fold increases in hardness due to suction. Notably, all of the measured data fell on a unique relationship: τ* = 1.02462*s*+0.17557 (r^2^ = 0.914, p<0.0001). Since the suction development of magnitude 2 kPa was less than the air-entry suctions for the sediments [19], such marked variation in the sediment hardness occurred in essentially saturated states of the intertidal flats and beaches that represented the habitats of *Ruditapes philippinarum* and *Donax semigranosus*.

In Figure 3B, the above field data are superimposed on the *s*–τ* relationships for the three different relative densities *D*_r_ = 40%, 60%, and 80% that were obtained in the laboratory for the sediments sampled at the Nojima flat. Note that the three different curves cover higher suction ranges than those shown in Figure 1D. The surficial intertidal sediments became denser at locations where higher suctions developed, yielding regions with *D*_r_>80%. In contrast, the sediments remained looser where low or negative suctions ensued, yielding regions with *D*_r_<40%.

By comparing Figures 1D and 3B, it is evident that all of the suctions *s*, relative densities *D*_r_, and sediment hardnesses τ* as simulated in the laboratory were in the ranges of the naturally varying geoenvironmental conditions in the field.

### Burrowing responses of *R. philippinarum* and *D. semigranosus*

**Figure 4:**
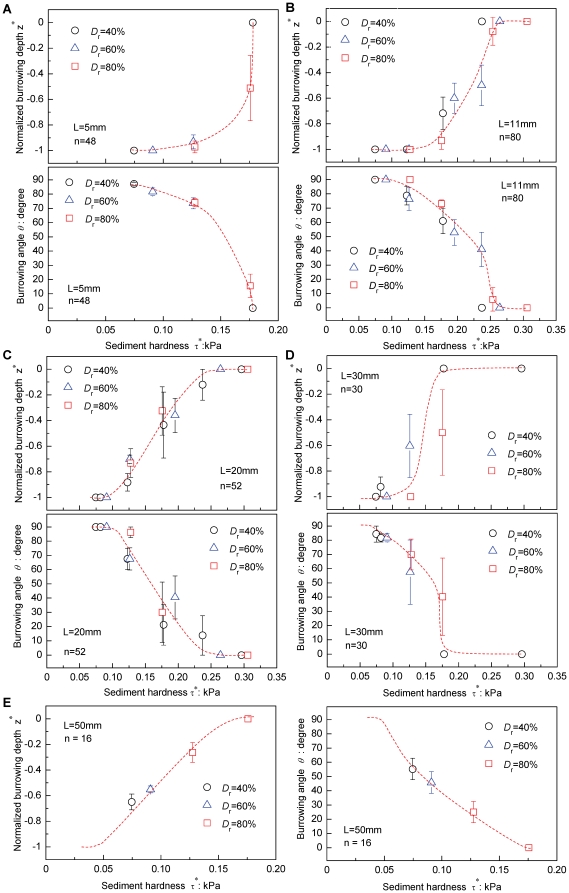
Burrowing criteria and burrowing mode adjustment in *Ruditapes philippinarum*. Measured normalized burrowing depth z^*^ and burrowing angle *θ* versus sediment hardness for five stages of growth: (A) L = 5 mm, (B) L = 11 mm, (C) L = 20 mm, (D) L = 30 mm, and (E) L = 50 mm. The sediment hardnesses τ* were varied by changing suctions *s* and sediment relative densities *D*_r_ at 40%, 60%, 80%, as shown in Table 1 and Figure 1D. Data represent mean values ± SE. The results of the related statistical analyses (GLM) are shown in Table 3A,B.

The observed responses of the juvenile to adult *R. philippinarum* to the varying hardness τ* (Table 1, Figure 1D) of the surficial intertidal sediments are summarized in Figure 4. In Figure 4A, all of the juvenile bivalves (L = 5 mm) completed vertical burrowing with *θ* = 90° and |*z* ^*^| = 1, at a low τ* = 0.07 kPa. However, with increasing τ*, the bivalves started to shift their burrowing modes, showing inclined complete burrowing, and then inclined partial burrowing. Indeed, the burrowing angle *θ* and the normalized burrowing depth |*z* ^*^| decreased significantly with increasing τ* (p<0.0001, Table 3A,B). At this stage, individual bivalves that failed to burrow in the sediment started to emerge (Table 1). Eventually, when τ* reached a certain value τ* = 0.18 kPa, all of the juvenile bivalves reached the non-burrowing state with *θ* = 0 and |*z* ^*^| = 0.

**Table 3:** Model selection results for (A) burrowing depth z^*^ and (B) burrowing angle *θ* on *Ruditapes philippinarum* and *Donax semigranosus*.

	A. Global model AIC: 201.47, best model AIC: 193.40				B. Global model AIC: 221.12, best model AIC: 216.48			
Explanatory variables	Estimate	SE	z value	p	Estimate	SE	z value	p
Intercept	−11.10	1.34	−8.273	<0.0001	9.53	1.16	8.222	<0.0001
Species	3.68	0.69	5.303	<0.0001	−3.35	0.64	−5.254	<0.0001
Shell length	0.07	0.02	4.382	<0.0001	−0.05	0.01	−3.402	0.0007
Sediment hardness	35.85	3.99	8.989	<0.0001	−32.17	3.54	−9.100	<0.0001

A generalized linear model (GLM) with a binomial error distribution to examine the effect of species, shell length, and sediment hardness on burrowing depth z* and burrowing angle *θ*. Only the best fitted models (the smallest AIC models) are shown.

The above results indicate that the burrowing mode shift occurred in a certain range of sediment hardnesses τ*. This means that there exist two burrowing criteria below or above which the bivalves accomplished vertical burrowing or failed to burrow, respectively. For the purpose of later discussion, we denote here the former and latter criteria by τ*_v_ and τ*_f_, respectively. Such burrowing criteria and burrowing mode adjustment can also be confirmed from the observed bivalve responses at different stages of growth (Figure 4B, C, D, E, Table 3A,B).

A notable difference is that both the normalized burrowing depth |*z* ^*^| and burrowing angle *θ* decreased significantly with increasing shell length L (p<0.001, Table 3A,B). Indeed, the adult bivalves (L = 30 mm and 50 mm) did not exhibit the vertical burrowing regime even under the lowest hardness, τ* = 0.07 kPa corresponding to loosely packed submerged sediments with *D*_r_ = 40% (Figure 1D). This indicates decreasing burrowing capability toward adult stages with increasing shell lengths (Table 3A,B).

**Figure 5:**
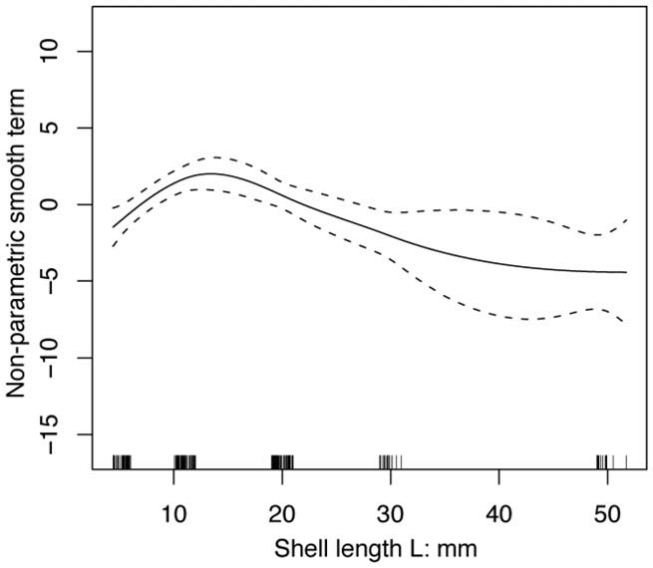
Results of statistical analysis (GAM) for the juvenile to adult *Ruditapes philippinarum*. A generalized additive model (GAM) with a binomial error distribution to examine how burrowing response co-varied with shell length and sediment hardness in *R. philippinarum*. The response variable for the model was binary data on vertical burrowing and burrowing mode shift (*a* and *b* in Table 1) and burrowing failure (*c* in Table 1). The solid and dotted lines represent the mean and 95% confidence intervals, respectively.

The results of the GAM analysis further showed that there exists a peak in the burrowing capabilities among the juvenile stages (Figure 5). Indeed, the juvenile bivalves with L = 10 to 20 mm achieved the highest burrowing capabilities of all growth stages for *Ruditapes philippinarum*.

**Figure 6:**
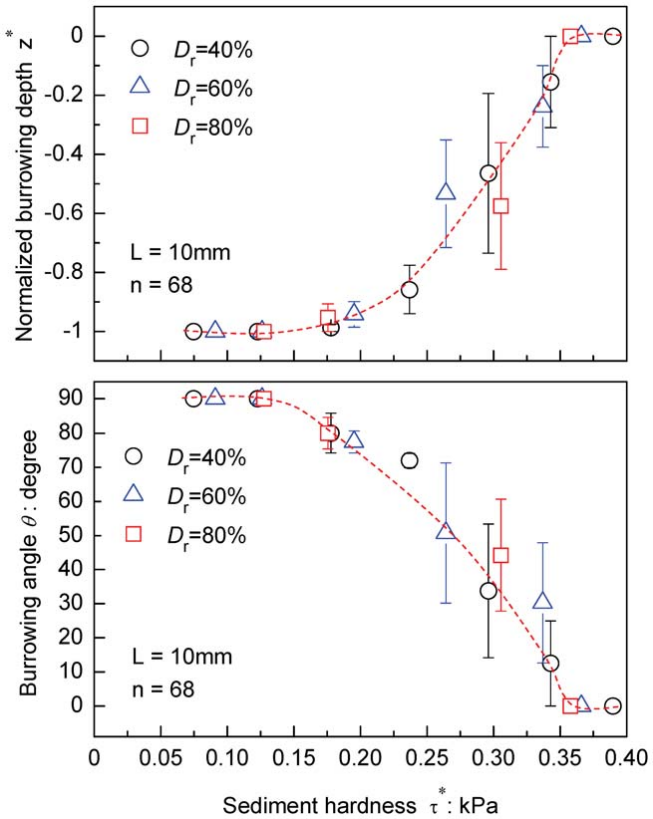
Burrowing criteria and burrowing mode adjustment in *Donax semigranosus*. Measured normalized burrowing depth z^*^ and burrowing angle *θ* versus sediment hardness for L = 10 mm. The sediment hardnesses τ* were varied by changing suctions *s* and sediment relative densities *D*_r_ at 40%, 60%, 80%, as shown in Table 2 and Figure 1D. Data represent mean values ± SE. The results of the related statistical analyses (GLM) are shown in Table 3A,B.

*Donax semigranosus* showed the same general burrowing characteristics in response to increasing hardness of the surficial intertidal sediments (Figure 6, Table 3A,B). Namely, there were three distinctive burrowing regions, namely, vertical burrowing, burrowing mode shift, and burrowing failure, depending on the magnitude of the sediment hardness τ*. Notably, both burrowing criteria τ*_v_ = 0.13 kPa and τ*_f_ = 0.36 kPa for *Donax semigranosus* were higher than those for all growth stages of *Ruditapes philippinarum*, by comparing Figures. 4 and 6, and also as shown in Tables 1–3.

## Discussion

### Mechanics of burrowing criteria and burrowing mode shift

**Figure 7:**
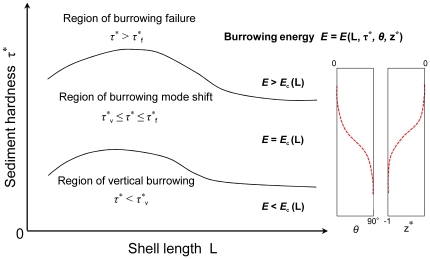
Energy-based conceptual model to account for the three distinctive burrowing regions for *Ruditapes philippinarum* and *Donax semigranosus*. For the bivalve with an individual traits, *E* denotes the required burrowing energy as a function of the shell length L, sediment hardness τ*, burrowing angle *θ* and normalized burrowing depth z^*^. *E*_c_ (L) denotes the burrowing capacity of the bivalve at a given growth stage. τ*_v_ represents the burrowing criteria below which the bivalve accomplishes vertical burrowing. τ*_f_ represents the burrowing criteria above which the bivalve fails to burrow.

The above results demonstrate that in both bivalves *R. philippinarum* and *Donax semigranosus*, there exist two burrowing criteria and burrowing mode adjustment to variations in hardness *τ* ^*^, as assessed by the vane shear strength, of the surficial intertidal sediments. To discuss the underlying mechanics further, we present a conceptual model based on consideration of the energy principle of bivalve burrowing (Figure 7). With reference to Figure 2, the bivalve burrowing consists of two processes, the swing-up from the sediment surface and the insertion of its body into the sediment. The required burrowing energy *E* for these two processes can be expressed in the following form:
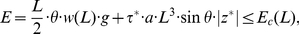
(4)where *a* is an intrinsic parameter pertaining to an individual traits, and *E_c_* (*L*) is the burrowing capacity, which essentially depends on the shell length *L*. The second term of eq. (4) stems from the concept of soil mechanics such that the soil resistance to insertion depends on the soil shear strength, and the depth and angle of insertion [39]. Eq. (4) tells us that the required energy for both the swing-up and insertion decrease with decreasing burrowing angle and depth (self-burial) under a given state *τ* ^*^.

In the vertical burrowing region (*τ* ^*^<*τ* ^*^_v_), the relationship 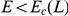 holds true, and thus the bivalve can complete the ideal vertical burrowing naturally within its own burrowing capacity. However, under situations where *τ* ^*^ exceeds the vertical burrowing region, the required burrowing energy reaches its capacity 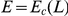, and therefore the bivalve tries to compensate for an excessive burrowing energy above its capacity by shifting its burrowing mode to the inclined mode in view of eq. (4). This means that the increase in *τ* ^*^ in this region (*τ* ^*^_v_≤*τ* ^*^≤*τ* ^*^_f_) yields continuous decreases in the burrowing angle *θ* and the normalized burrowing depth |z*|. Also, as the maximum burrowing capacity has already been reached in this region, there starts to occur a selection of individuals that can and cannot afford to undergo the burrowing mode shift, where the latter leads to a non-burrowing state. Eventually, insertion of the foot itself becomes impossible in the region of burrowing failure (*τ* ^*^>*τ* ^*^_f_).

The above discussions suggest that, even in the vertical burrowing region, the bivalves may take an option of undergoing the inclined burrowing mode due to its lower energy expenditure, subsequently making their bodies upright in the sediment. However, this post-uprighting process requires them to push the sediment laterally as well as upward and to overcome the accompanying high passive earth pressure due to sediment pushing [39]. Therefore, the bivalves should undergo vertical burrowing to reach the stable vertical position in the sediment.

Since insertion of the body with a lower apex angle requires less energy at a given burial depth in the sediment [43], the value of the parameter *a* in eq. (4) becomes lower for a sharper apex, such as that of *Donax semigranosus*, than for a more round apex, such as that of *R. philippinarum* (Figure 2). This means that *D. semigranosus* reaches its burrowing capacity at higher hardness τ* than *R. philippinarum* does, giving rise to the higher burrowing criteria for *D. semigranosus*.

Overall, this conceptual model is capable of consistently accounting for the observed bivalve responses, namely, the manifestations of vertical burrowing, burrowing mode shift, and burrowing failure, in response to the change in hardness due to the varying geoenvironmental conditions.

### Role and implications of burrowing criteria and burrowing mode shift

The surficial sediments of intertidal flats and beaches exhibited distinct variations in hardness due to the effects of suction dynamics that represent suction development and suction dynamics-induced sediment compaction [19] (Figure 3). The simulated geophysical environmental conditions involving suction, relative density, and hardness of the surficial intertidal sediments were all realistic values seen in the field. Notably, both *R. philippinarum* and *Donax semigranosus* showed acute sensitivities in their responses, giving rise to the transitions between vertical burrowing, burrowing mode shift, and burrowing failure, to variations in such prevailing geoenvironmental conditions. Under conditions where burrowing fails to take place, the bivalves become exposed at the sediment surface. Hence, they can be easily transported away offshore or onshore by waves and currents [31] and are exposed to fatal risks from predators [32] and also from direct rays of the sun [33], all of which reduce the chances of survival.

The burrowing mode shift shows a distinct ability of the bivalves to adapt to a harder geophysical environment. Indeed, the combined results from the experiments, field surveys, and the conceptual model demonstrate that the bivalves assess the hardness of the surficial sediments and sensibly adjust their burrowing modes in order to cope with a hardness greater than what they can cope with in their normal vertical modes. The inclined burrowing that enables complete burial should substantially reduce the risk of being caught by predators or washed away when they persist in the normal vertical mode and become exposed under such severe geoenvironmental conditions.

With given siphon sizes, however, inclined burrowing means shallower burial depth, which decreases the chance of survival [29], compared with vertical burrowing, which assures an ideal stable position in the sediment in terms of feeding strategy and resistance against hydrodynamic forcing. Furthermore, with increasing hardness, inclined burrowing is accompanied by selection of individuals exhibiting partial burrowing and burrowing failure (Tables 1 and 2). Hence, vertical burrowing conditions may represent suitable geoenvironmental conditions for burrowing, and thus for their survival.

Bivalves, as suspension-feeders, consume oxygen and food from the overlying water, whose availability may depend on the hydroenvironmental conditions. Here, we show that the bivalve responses to the varying geoenvironmental conditions have important implications for the adult–juvenile spatial distributions of *R. philippinarum*. Namely, for the adult *R. philippinarum*, the suitable vertical burrowing conditions represented looser states of submerged sediments with *D*_r_<40%. In contrast, the burrowing capability increased considerably toward juvenile stages of *R. philippinarum*, who accomplished vertical burrowing even under the densest state of submerged sediments with *D*_r_ = 80% (Tables 1 and 3A,B). This marked contrast may elucidate the mechanism underlying the observed cross-shore distributions of adult *R. philippinarum* that were limited to the lower intertidal zones (Shirakawa tidal flat, [35]) where the sediments remained loose with *D*_r_<40% under negative suctions (Figure 3B), whereas juveniles exhibited much wider distributions in the entire range of intertidal zones involving denser sediments with *D*_r_≈80% ([35], Figure 3B).

Similarly, in bar–trough intertidal sediments, suction development and suction dynamics-induced sediment compaction at the bar [19] hinder effective burrowing for all sizes, particularly adult *R. philippinarum* (Table 1, Figure 4). This can account for the popular concentrated shellfish gathering at the water's edge, where the sediments remain loose due to the absence of suction, yet are dynamically more stable than the adjacent troughs as part of the persistent sandbars [26], thereby preserving a suitable geoenvironment for the burrowing throughout the course of the tides. This may lead to an effective habitat design for *R. philippinarum* in the framework of ecological restoration [44].

Also, once the bivalve is brought to the sediment surface due to hydrodynamic forcings, bioturbation, or human disturbances, whether or not the bivalve can resettle in the sediment depends on the balance between the changing burrowing capabilities and the ensuing geoenvironment. Such a balance could have a long-lasting impact on the life cycle of *R. philippinarum* from juvenile to adult in given particular habitats.

From conservational and fishery points of view, recruitment of post-larval *R. philippinarum* can be enhanced by sediment reworking because the burrowing capability is low until the juvenile stage (Figure 5). This would be effectively done with the management of suction, which is closely linked with micro-topography and groundwater level [19], since the hardness variation is essentially brought about by suction (Figure 3).

**Figure 8:**
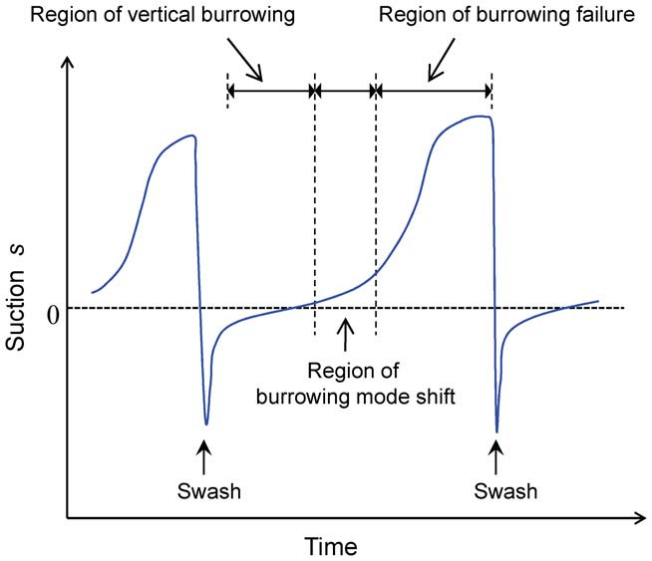
Sketch showing the measured relationships between swash-induced suction dynamics and three burrowing regions for *Donax semigranosus*. The average swash intervals observed during the spring low tides at Yuigahama and Kujyukuri beaches ranged from 20 s to 10 min. When swash retreated in the course of the swash repetitions, suction increased to 2 kPa, corresponding to a 20- to 50-fold increase in the sediment hardness at the sandy beach habitats (Figure 3A).

The burrowing criteria and burrowing mode shift manifested in *D. semigranosus* at higher geoenvironmental ranges than all sizes of *R. philippinarum*. Also, *D. semigranosus* burrowed faster than all sizes of *R. philippinarum*. These may reflect the adaptations of *D. semigranosus* to the geoenvironmental changes that occur more rapidly and severely in intertidal zones of exposed sandy beaches than in intertidal sandy flats. Indeed, the suction dynamics at sandy beach habitats is brought about by the swash in addition to the tide (Figure 8). *Donax* species are well-known for their rapid burrowing behavior, which is considered to be governed by the swash climates under given grain sizes [8],[11],[12],[25]. That is to say, the bivalves that have migrated on a given swash need to burrow rapidly in order to escape from the wash out due to the next swash. However, our field surveys combined with the experimental results demonstrated that between each swash, which had intervals ranging from 20 s to 10 min, the groundwater level fluctuated [45],[46], causing suction dynamics that gave rise to the burrowing failure regime (Figure 8). In fact, during the course of the swash repetitions, suction increased to *s* = 2 kPa, which corresponded to a 20- to 50-fold increase in hardness at the sandy beach habitats (Figures 3A and 8). In contrast, *D. semigranosus* could not burrow when the groundwater level became slightly below the sediment surface such that z = −7 mm with *s* = 0.07 kPa for *D*_r_ = 80% and z = −17 mm with *s* = 0.17 kPa for *D*_r_ = 60% that gave rise to the same burrowing criteria τ*_f_ = 0.36 kPa (Table 2, Figure 6). The above results clearly indicate that the bivalves need to burrow rapidly not to escape from the next swash, but to avoid the preceding rapidly increasing hardness of the surficial intertidal sediments, making burrowing physically impossible (Figure 8). This demonstrates a new mechanism for their behavioral adaptation as governed by the rapidly changing geoenvironment at beaches. The preceding discussion of their traits further implies that the streamlined shape of *Donax* may also be a result of the morphological adaptation to the severer geoenvironmental changes ensuing at the beaches.

The present study has revealed two new burrowing criteria and burrowing mode adjustment to varying geoenvironmental conditions in the two different species of bivalves, *R. philippinarum* and *D. semigranosus*. It also highlighted their mechanics, as well as their role and implications, in the adult–juvenile spatial distributions and behavioral and morphological adaptations at the intertidal flats and beaches. Ongoing and future sea level rises will change the state of long-term groundwater level fluctuations and thus suction dynamics, relative density, and hardness of the surficial intertidal sediments. On the basis of the integrity of the responses of the bivalves to varying geoenvironmental conditions, the results and discussions presented in this paper may serve as a basis of not only the ensuing but also the future bivalve responses to geoenvironmental changes in intertidal zones.
